# Ice Houses

**Published:** 1873-02

**Authors:** 


### ICE HOUSES

Are becoming almost as indispensable to families as the old-fashioned smoke house. It does not add to the healthfulness of any liquid to be made ice cold, especially at meals. Those are best off, who are satisfied with water as it comes from the well or spring or croton pipe, the year round, but it is valuable as a food preserver, and is a remedial agent of great importance in all forms of fever, inflammations, croup, diarrhœa, and dysentery, as is more fully discussed in our last work, "Hall's Family Doctor."

The best place to build an ice house is on a slight descent, and on the north side of a hill, but level ground will answer. Set posts or unhewed logs into the ground two feet deep, and a foot and a half apart, twelve feet square, and twelve feet high, and board it up on the inside; arrange another set of posts fourteen feet square, on the outside of the other, and board it up on the outside. Have a plank all around between these, about half a foot from the ground, and fill up with very dry tan bark or saw-dust; make an ordinary four-square slanting roof to each set of posts, or a flat roof, the space in either case to be filled with tan or saw-dust. Have a door on the north side, have some logs on the bottom a few inches apart, lay some planks loosely across these logs, scatter over a foot or more of straw, lay in your ice as close as you can, dig a gutter all around on the outside a foot deep, and a foot or two from the outer planks, and throw up the dirt so as to form a bank against the bottom of the outer planks, so arranging everything, that the water from the ice or the weather shall have a free outlet on the lower side of the hill. Such a house will keep ice the year round for any two ordinary families. It was once thought that a hole should be left in the roof of an ice house for ventilating purposes; this is not the case.

